# Deducing the Role of Virus Genome-Derived PIWI-Associated RNAs in the Mosquito–Arbovirus Arms Race

**DOI:** 10.3389/fgene.2019.01114

**Published:** 2019-11-28

**Authors:** Carol D. Blair

**Affiliations:** Arthropod-borne and Infectious Diseases Laboratory, Department of Microbiology, Immunology and Pathology, Colorado State University, Fort Collins, CO, United States

**Keywords:** arbovirus, Aedes mosquitoes, PIWI-associated RNA, RNA interference, endogenous viral elements

## Abstract

The P-element-induced wimpy testis (PIWI)-associated RNA (piRNA) pathway is known for its role in the protection of genome integrity in the germline of *Drosophila melanogaster* by silencing transposable elements. The piRNAs that target transposons originate from piRNA clusters in transposon-rich regions of the *Drosophila* genome and are processed by three PIWI family proteins. In *Aedes aegypti* and *Aedes albopictus* mosquitoes, which are two of the most important vectors of arthropod-borne viruses (arboviruses), the number of PIWI family genes has expanded and some are expressed in somatic, as well as germline, tissues. These discoveries have led to active research to explore the possible expanded functional roles of the piRNA pathway in vector mosquitoes. Virus genome-derived piRNAs (which will be referred to as (virus name) vpiRNAs) have been demonstrated in *Aedes* spp. cultured cells and mosquitoes after infection by arthropod-borne alpha-, bunya-, and flaviviruses. However, although *Culex quinquefasciatus* also is an important arbovirus vector and has an expansion of PIWI family genes, vpiRNAs have seldom been documented in this genus after arbovirus infection. Generation of complementary DNA (cDNA) fragments from RNA genomes of alpha-, bunya-, and flaviviruses (viral-derived cDNAs, vDNAs) has been demonstrated in cultured *Aedes* spp. cells and mosquitoes, and endogenous viral elements (EVEs), cDNA fragments of non-retroviral RNA virus genomes, are found more abundantly in genomes of *Ae. aegypti* and *Ae. albopictus* than other vector mosquitoes. These observations have led to speculation that vDNAs are integrated into vector genomes to form EVEs, which serve as templates for the transcription of antiviral vpiRNA precursors. However, no EVEs derived from alphavirus genomes have been demonstrated in genomes of any vector mosquito. In addition, although EVEs have been shown to be a source of piRNAs, the preponderance of EVEs described in *Aedes* spp. vectors are more closely related to the genomes of persistently infecting insect-specific viruses than to acutely infecting arboviruses. Furthermore, the signature patterns of the “ping-pong” amplification cycle that maintains transposon-targeting piRNAs in *Drosophila* are also evident in alphavirus and bunyavirus vpiRNAs, but not in vpiRNAs of flaviviruses. These divergent observations have rendered deciphering the mechanism(s) of biogenesis and potential role of vpiRNAs in the mosquito–arbovirus arms race difficult, and the focus of this review will be to assemble major findings regarding vpiRNAs and antiviral immunity in the important arbovirus vectors from *Aedes* and *Culex* genera.

## Introduction

Arthropod-borne viruses (arboviruses) can cause devastating and sometimes deadly diseases when they are transmitted to humans and other vertebrates, but for the most part their infection of their arthropod vectors does not result in discernable fitness loss (Putnam and Scott, 1995; Lambrechts and Scott, 2009). This seemingly paradoxical phenomenon has been attributed partially to the divergent antiviral innate immune responses of the two phylogenetically diverse natural hosts (Blair, 2011).

We began to understand the unique RNA-based mosquito innate immune system when we emulated research published by plant virologists showing that expression in the cytoplasm of transgenic plants of a non-translatable segment of a plant virus RNA genome resulted in inhibition of homologous virus replication upon challenge (Lindbo and Dougherty, 1992; Ratcliff et al., 1997). We showed that expression of a 500-nt segment of dengue virus type 2 (DENV2, *Flavivirus*) RNA under control of the second subgenomic promoter of a recombinant Sindbis virus (SINV, *Alphavirus*) could block DENV2 replication in *Aedes aegypti* mosquitoes (Olson et al., 1996) as well as *Aedes albopictus* (C6/36) mosquito cells infected by the recombinant virus (Gaines et al., 1996). We further showed that C6/36 mosquito cells could be rendered heritably resistant to replication of DENV2 by transformation with a plasmid that expressed an inverted repeat segment derived from the DENV2 genome (Adelman et al., 2002).

When fly researchers determined that injection into *Drosophila melanogaster* embryos of double-strand RNA (dsRNA) derived from a *Drosophila* gene resulted in silencing of expression of that gene (Kennerdell and Carthew, 1998), we hypothesized that expression in mosquitoes of dsRNA derived from the DENV genome resulted in silencing of expression of the DENV genome. We later concluded that viral dsRNA was the activator of mosquito antiviral immunity (Keene et al., 2004; Sánchez-Vargas et al., 2009). (Our subsequent discovery that C6/36 cells have a defect in their *dicer2* gene, which encodes a key enzyme in the dsRNA-based immunity pathway (Scott et al., 2010), led to an extension of our hypothesis, which will be expanded upon below and is the subject of this review.)

Since *Drosophila* and mosquitoes are members of the same order (Diptera), extensive discoveries in *D. melanogaster* (Ghildiyal and Zamore, 2009) were frequently used as models in our studies. We and others pursued definition of the *Ae. aegypti* RNA interference (RNAi) pathway involved in antiviral immunity (Franz et al., 2006; Sánchez-Vargas et al., 2009), which was distinguished from other small RNA (sRNA) silencing pathways by the designation exogenous-small interfering RNA (exo-siRNA).

As complete mosquito genome sequences became available (Holt et al., 2002; Nene et al., 2007), we were able to demonstrate that *Drosophila*-orthologous mosquito genes and their encoded proteins were involved in the antiviral exo-siRNA pathways of *Anopheles gambiae* (Keene et al., 2004) and *Ae. aegypti* (Sánchez-Vargas et al., 2009). Major exo-siRNA pathway proteins with orthologues in mosquitoes (Campbell et al., 2008) were the RNase III family endonuclease Dicer 2 (Dcr2) (Bernstein et al., 2001), the dsRNA-binding protein R2D2 (Liu et al., 2003), and the ssRNA endonuclease Argonaute 2 (Ago2) (Rand et al., 2004; van Rij et al., 2006).

Our early RNAi studies in C6/36 cells engineered to express dsRNA derived from the DENV2 genome showed that sRNAs generated from the dsRNA sequence migrated between 20- and 30-nt size markers in northern blots, with a size appearing to be larger than 21 nt (Adelman et al., 2002). The cells expressing these sRNAs were resistant to DENV2 infection; however, we subsequently discovered that C6/36 cells are deficient in Dcr2 activity, and thus unable to process long dsRNA into 21-nt exo-siRNA. Because we found that C6/36 cells also produce more abundant virus genome-derived P-element-induced wimpy testis-interacting (PIWI) RNA (vpiRNA)-like sRNA than Dcr2-competent *Ae. aegypti* cells following arbovirus infection (Brackney et al., 2010; Scott et al., 2010), we speculated that in exo-siRNA-defective *Aedes* spp. mosquito cells, the PIWI-interacting RNA (piRNA) response serves a compensatory, although less effective, antiviral function. It was later shown that exo-siRNA pathway deficiencies also occur in *Ae. albopictus* C7-10 cells (Morazzani et al., 2012) and *Aedes pseudoscutellaris* AP-61 cells (Göertz et al., 2019), with apparent compensatory vpiRNA production following arbovirus infection.

Arbovirus vpiRNAs have frequently been found to occur in addition to exo-siRNAs in *Aedes* spp. mosquito cells infected with alpha-, bunya-, and flaviviruses. Although *Culex* spp. mosquitoes are also important arbovirus vectors, recent research indicates arbovirus infection of *Culex* mosquitoes and cells much less frequently induces vpiRNAs (Göertz et al., 2019; Rückert et al., 2019).

Observations of vpiRNAs in some exo-siRNA-competent arbovirus-infected mosquito cells have spurred increased research to answer the question: What role, if any, do vpiRNAs play in the vector mosquito RNA-mediated antiviral response? Reviews of the limited research on this subject have attempted to construct a coherent picture while pointing out the many questions that remain to be explored (Miesen et al., 2016b; Varjak et al., 2018). Also, a recent review described expanded PIWI family genes and vpiRNAs in other insects (Kolliopoulou et al., 2019). However, results from studies targeting arboviruses from various families, mosquitoes and mosquito cell cultures from different genera, and different experimental approaches, have yielded apparently inconsistent, divergent, even discordant results. Since research to date is far from comprehensive, my focus will necessarily be on the major vectors of *Aedes* spp., but will contrast *Culex* where research has been done, and on arboviruses that are major disease problems or “model organisms.” I will try to capture conclusions from studies to date by posing a series of questions underlying the research and will attempt to indicate additional research that might fill in gaps in our understanding.

## Do Arboviruses and Insect-Specific Viruses Representing Varying Families, or Even Different Species Within the Same Family, All Elicit Production of vpiRNAs During Infection of Natural Vector Mosquitoes? and Do All *Aedes* and *Culex* Spp. Vector Mosquitoes That Have Been Examined Produce vpiRNAs in Response to Arbovirus Infections?

In *Drosophila*, the piRNA system acts through three major proteins, Piwi, Aubergine (Aub), and Argonaute 3 (Ago3), and has the primary function of defending the genome against transposable element activity. These proteins are expressed only in germline and associated cells. Piwi and Aub bind piRNA precursor RNAs transcribed from piRNA clusters in the genome and process them to (usually) negative-sense 25- to 30-nt piRNAs that have a bias for a uridine base at the 5′ end (U1) (Brennecke et al., 2007). Aub-bound primary piRNAs in the cytoplasm hybridize to complementary sequences in transposon transcripts with a 5′-end 10-nt offset and are cleaved by Aub to produce secondary piRNAs, then bound to Ago3 (Brennecke et al., 2007; Gunawardane et al., 2007).

The Ago3-bound piRNAs are positive-sense polarity with adenine in position 10 (A10). These interactions initiate the “ping-pong” cycle, in which the Ago3-bound secondary piRNA hybridizes to a complementary piRNA cluster-derived transcript, which is cleaved to produce a new piRNA and perpetuate the cyclic production of transposon-targeting piRNAs (Brennecke et al., 2007; Gunawardane et al., 2007). The “ping-pong signature” thus consists of a population of 25- to 30-nt piRNAs that are negative-sense, U1-biased, and positive-sense A10-biased with complementarity offset by 10 nt.

Although vpiRNAs were first recognized in a *Drosophila* ovary somatic sheet (OSS) cell line that was persistently-infected with three RNA viruses (Wu et al., 2010), we now know that the *Drosophila* model for studies of potential antiviral functions of the piRNA pathway in subfamily Culicinae mosquitoes (*Aedes* and *Culex* spp.) is limited. Although flies express their three PIWI family genes only in germline and associated tissues, the PIWI family has expanded to seven or eight genes in *Ae. aegypti, Ae. albopictus*, and *Culex pipiens* mosquitoes (Campbell et al., 2008), at least four of which (Ago 3 and Piwi 4, 5, and 6) are expressed in somatic tissues of *Ae. aegypti* (Akbari et al., 2013; Miesen et al., 2015). Further, it has been found that vpiRNAs are not produced in adult flies and the piRNA pathway does not play a major role in antiviral defense in adult *Drosophila* (Petit et al., 2016).

*Ae. aegypti* and *Ae. albopictus* are predominant vectors of human–pathogenic arboviruses, largely because of their widespread geographic distributions, habitats reflecting their anthropophilic feeding habits, and increasing invasion of new regions (Weaver and Reisen, 2010). One or both of these species transmits numerous currently circulating arboviruses that cause diseases of public health concern, most of which are members of the families *Flaviviridae*, *Togaviridae*, and *Peribunyaviridae* and *Phenuiviridae* of the order *Bunyavirales* (International Committee on Taxonomy of Viruses, https://talk.ictvonline.org/ictv-reports/ictv_online_report/). Arboviruses, by definition, are maintained in nature in a horizontal transmission cycle in which they replicate in both the arthropod vector and vertebrate host.

Mosquito-specific or insect-specific viruses (ISVs), which frequently are shown to resemble arboviruses in structure, genome organization, and nucleotide sequence, are unable to infect and replicate in vertebrates, and their widespread distribution and high abundance in *Aedes* spp. and *Culex* spp. vector mosquitoes have been increasingly noted over the past two decades. Their maintenance in nature, where investigated, is by vertical transmission from mother to offspring (Lutomiah et al., 2007; Saiyasombat et al., 2011; Bolling et al., 2012; Haddow et al., 2013). ISVs have been described that can be classified in each of the four taxa above by phylogenetic analyses, as well as in the families *Rhabdoviridae, Reoviridae, Mesoniviridae, Tymoviridae*, and *Birnaviridae* (Bolling et al., 2015).

Researchers who carried out deep sequencing of small RNAs in mechanistic studies of the major RNA-based antiviral response, exo-siRNA, were among the first to observe that arbovirus genome-derived vpiRNAs as well as exo-siRNAs occurred in flavivirus (DENV2)-infected *Aedes* spp. mosquitoes and cultured cells following exogenous infection (Scott et al., 2010; Hess et al., 2011). Other studies followed to show that alphavirus, bunyavirus, orbivirus, and other flavivirus acute infections of *Aedes* spp. cells also elicited biogenesis of vpiRNAs (Brackney et al., 2010; Morazzani et al., 2012; Vodovar et al., 2012; Léger et al., 2013; Schnettler et al., 2013; Aguiar et al., 2015; Miesen et al., 2015; Goic et al., 2016; Dietrich et al., 2017; see Miesen et al., 2016b for review) (see **Table 1**).

**Table 1:** Arboviruses and insect-specific viruses (ISVs) shown to elicit biogenesis of virus genome-derived piRNAs (vpiRNAs) in infected *Aedes* spp. mosquito vectors.

Virus order/family/genus	Virus name	Host mosquito/cell culture	vpiRNA polarity/nucleotide bias	vDNA demonstrated?
*Togaviridae/Alphavirus*	SINV	Aag2, U4.4, C6/36	neg.-pos./U1-A10	
	SFV	Aag2, U4.4	neg.-pos./U1-A10	
	CHIKV	*Ae. aegypti, Ae albopictus,* U4.4,C6/36, C7-10	neg.-pos./U1-A10	Yes
*Flaviviridae/Flavivirus*	DENV2	*Ae. aegypti*, Aag2, C6/36	pos./A10	Yes
	ZIKV	Aag2, Aag	pos./A10	Yes
	WNV	Aag2, C6/36, U4.4, AP-61	pos./A10	Yes (C6/36, Aag2)
	CFAV	Aag2, C6/36	pos./A10	
*Bunyavirales/Peribunyaviridae/Orthobunyavirus*	LACV	C6/36	neg.-pos./U1-A10	Yes
*Bunyavirales/Phenuiviridae/Phlebovirus*	RVFV	Aag2, U4.4, C6/36	neg.-pos./U1-A10	
*Bunyavirales/Phenuiviridae/Phasivirus*	PCLV	*Ae. aegypti,* Aag2	neg.-pos./U1-A10	No
*Birnaviridae/Entomobirnavirus*	CYV	Aag2, U4.4, C7-10	neg.-pos. (C7-10 only)	Yes (U4.4)

CFAV, cell fusing agent virus; CHIKV, chikungunya virus; CYV, Culex Y virus; DENV2, dengue virus serotype 2; LACV, La Crosse virus; PCLV, phasi charoen-like virus; RVFV, Rift Valley fever virus; SFV, Semliki Forest virus; SINV, Sindbis virus; WNV, West Nile virus; ZIKV, Zika virus.

Interestingly, *Culex* spp. mosquitoes and cultured cells, although also shown to have an expanded PIWI gene family (Campbell et al., 2008), have been consistently shown not to produce vpiRNAs as a result of acute arbovirus infection (Brackney et al., 2009; Fros et al., 2015; Miesen et al., 2016b; Rückert et al., 2019; Göertz et al., 2019). For example, although the flavivirus West Nile virus (WNV) elicited production of vpiRNAs in cultured *Aedes* spp. cells (Aag2 and U4.4) (Göertz et al., 2019), WNV-specific vpiRNAs were not observed in the midgut after acute infection of its natural vector *Culex quinquefasciatus* mosquitoes (Brackney et al., 2009) or *Culex* spp. cell cultures (CT, Hsu) (Göertz et al., 2019; Rückert et al., 2019). However, low levels of Rift Valley fever virus (RVFV, *Phenuiviridae*) vpiRNAs were detected in whole bodies (presumably containing germline tissue) of acutely infected *Cx. quinquefasciatus* mosquitoes (Dietrich et al., 2017).

Both *Aedes* spp. and *Culex* spp. mosquito cell cultures are frequently persistently infected with ISVs, and, in most cases, vpiRNAs derived from ISV genomes can be found in both *Culex-* and *Aedes-*derived cultured cells (Göertz et al., 2019; Rückert et al., 2019). For example, Hsu cell cultures [derived from *Cx. quinquefasciatus* ovary cells (Hsu et al., 1970)] that are persistently infected with Merida virus (MERV, *Rhabdoviridae*) (Weger-Lucarelli et al., 2018) were shown to generate MERV-specific vpiRNAs (Rückert et al., 2019) (see **Tables 1** and **2**).

**Table 2:** Arboviruses and insect-specific viruses (ISVs) for which biogenesis of virus genome-derived piRNAs (vpiRNAs) has been sought in infected *Culex* spp. mosquito vectors.

Virus order/family/genus	Virus name	Host mosquito/cell culture	vpiRNA polarity/nucleotide bias	vDNA demonstrated?
*Flaviviridae/Flavivirus*	WNV	*Cx. quinquefasciatus*, CT, Hsu	No	Yes (CT and Hsu only)
	CLBOV	CT	No	
*Togaviridae/Alphavirus*	SINV	*Cx. pipiens*	No	
*Bunyavirales/Phenuiviridae/Phlebovirus*	RVFV	*Cx. quinquefasciatus*	Yes neg.-pos./U1-A10	
*Bunyavirales/Phenuiviridae/Phasivirus*	PCLV	CT	Yes neg.-pos./U1-A10	
*Rhabdoviridae*	MERV	Hsu	Yes neg.-pos./U1-A10	Yes

CLBOV, Calbertado virus; MERV, Merida virus; PCLV, phasi charoen-like virus; RVFV, Rift Valley fever virus; SINV, Sindbis virus; WNV, West Nile virus.

To summarize, a number of investigations have shown that acute infections with alpha-, flavi-, and bunyaviruses or persistent infections with various ISVs consistently generate vpiRNAs in *Aedes* spp. mosquitoes and cell cultures derived from developmental stages of these mosquitoes. Naturally occurring ISV infections of cultured *Culex* spp. mosquito cells can also result in vpiRNA production; however, *Culex* spp. mosquitoes frequently have been found not to produce vpiRNAs after acute arbovirus infection.

### Is the vpiRNA Response of a Particular Vector Genus to Arboviruses of Different Families the Same?

Infection of *Aedes* spp. mosquitoes and cell cultures derived from developmental stages of these mosquitoes with either flaviviruses or alphaviruses that are naturally vectored by that particular mosquito presents an interesting contrast.

In chikungunya virus (CHIKV, *Alphavirus*)-infected *Ae. aegypti* and *Ae. albopictus* mosquitoes and *Ae. albopictus* U4.4, C6/36, and C7-10 cultured cells, in SINV (*Alphavirus*)-infected *Ae. aegypti* Aag2 cell cultures, and in Semliki Forest virus (SFV, *Alphavirus*)-infected Aag2 cells, abundant vpiRNAs with both genomic (positive-sense) and anti-genomic polarity and bearing the ping-pong signature (anti-genomic U1-genomic A10) are produced (Morazzani et al., 2012; Miesen et al., 2015; Varjak et al., 2017a). Infection of *Ae. albopictus* C6/36 or U4.4 or *Ae. aegypti* Aag2 cells with the tripartite, negative-sense RNA bunyaviruses La Crosse (LACV) or RVFV elicits a vpiRNA response similar to alphaviruses, with genomic and anti-genomic vpiRNAs bearing the ping-pong signature (Vodovar et al., 2012; Léger et al., 2013).

On the other hand, C6/36 cells, Aag2 cells, and *Ae. aegypti* mosquitoes, following infection with DENVs (*Flavivirus*), produce far less abundant vpiRNAs than during alphavirus or bunyavirus infections, with almost exclusively genomic (positive-sense) polarity and an A10 nucleotide bias (Scott et al., 2010; Miesen et al., 2016a). Although there has been some speculation that 27-nt sRNAs lacking a ping-pong signature in infected cells might be viral genome degradation products (Brackney et al., 2010; Rückert et al., 2019), these vpiRNAs have a distinct pattern closely resembling those produced in *Drosophila* OSS cells that express only the Piwi protein (Wu et al., 2010).

Thus, the same *Aedes* spp. mosquitoes and cultured cells exhibit different vpiRNA responses to arboviruses from different families with different genome structures and replication patterns.

### Is the vpiRNA Response of Different Vector Mosquito Genera to the Same Arbovirus or Arbovirus Family the Same?

As mentioned above, WNV (*Flavivirus*) infection presents an interesting example of the differing sRNA responses of *Culex* and *Aedes* spp. mosquitoes. WNV is normally vectored by *Culex* spp. mosquitoes (e.g., *Cx. quinquefasciatus, Cx. pipiens,* *Culex tarsalis*), but *Aedes* spp. mosquitoes can also be competent vectors (Turell et al., 2001). Recent investigations of the ability of *Cx. quinquefasciatus* mosquitoes and *Culex* spp.-derived cell cultures to generate WNV vpiRNAs after infection have determined that *Cx. quinquefasciatus* midguts and salivary glands, CT (*Cx. tarsalis*) and Hsu (*Cx. quinquefasciatus* ovary) cells did not form vpiRNAs (Rückert et al., 2019), confirming earlier studies with *Cx. quinquefasciatus* mosquitoes (Brackney et al., 2009). Other recent studies showed that WNV vpiRNAs were formed in the *Aedes* spp. cell cultures U4.4, Aag2, C6/36, and AP-61 (Göertz et al., 2019), although, importantly, only very low levels of 26- and 28-nt WNV-specific vpiRNAs with possible A10 sequence bias were detected in *Ae. aegypti* midguts after infection (Rückert et al., 2019). In confirmation of differing responses in the two mosquito genera, Miesen et al. (2016b) showed that no SINV (*Alphavirus*)-specific vpiRNAs were detected in SINV-infected *Cx. pipiens* mosquitoes, in stark contrast to the abundant responses to alphavirus infection of *Aedes* spp.-derived U4.4 and Aag2 cells (Vodovar et al., 2012; Miesen et al., 2015; Varjak et al., 2017b).

*Anopheles* spp. mosquitoes are major vectors of malaria parasites, but are known to transmit only one arbovirus, the alphavirus O’nyong-nyong (Williams et al., 1965). Because the complete genome sequence of *An. gambiae* was the first mosquito sequence to be published (Holt et al., 2002), we used O’nyong-nyong virus (ONNV)-infected *An. gambiae* to identify the genes involved in the RNAi response (Keene et al., 2004). We found that RNAi-mediated knockdown of both AgAgo2 and AgAgo3 resulted in significantly increased replication of ONNV in *An. gambiae*. In a study characterizing the ONNV genome-specific small RNAs in the midgut of infected *An. gambiae*, the major response was found to be 21-nt exo-siRNAs. A small peak of 27-nt ONNV-specific RNA was detected, but not further characterized (Carissimo et al., 2015). Unlike the mosquitoes of subfamily Culicinae, the PIWI family genes of *An. gambiae* have not expanded beyond the number found in *Drosophila* (Campbell et al., 2008). Whether PIWI family genes of *An. gambiae* are expressed outside the germline tissue and contribute to a vpiRNA-like response to arbovirus infection remains to be determined.

Research relevant to differences in the vpiRNA response in different vector genera has shown that Ago3 and Piwi4, Piwi5, and Piwi6 were expressed in *Ae. aegypti* midguts (Akbari et al., 2013; Miesen et al., 2016b), while low levels (in comparison to ovary expression levels) of Ago3, Piwi2, and Piwi5 were expressed in *Cx. quinquefasciatus* midguts (Rückert et al., 2019).

In summary, it is clear that not only can the piRNA responses of a particular mosquito genus be different when infected with arboviruses of different families, but also the responses to a particular arbovirus or arbovirus family by different mosquito genera can differ. Overall, studies conducted to date suggest that mosquito cell vpiRNA responses are specific to each arbovirus–mosquito combination.

## What Is the Mechanism for Biosynthesis of vpiRNAs in Vector Mosquitoes?

While comparing the transposon-specific piRNAs in *Ae. aegypti* with those in *Drosophila*, Arensburger et al. (2011) noted that a substantial proportion of the piRNA-generating DNA sequences in *Ae. aegypti* were of viral origin; that is, genomes of five different lines of *Ae. aegypti* mosquitoes contained virus-like sequences that were templates for piRNA precursor transcription. A number of subsequent studies have explored the endogenous viral elements (EVEs) or non-retroviral integrated RNA virus sequences (NIRVS) of the *Ae. aegypti* and *Ae. albopictus* genomes, noting that the proportion of these genomes occupied by EVEs is far greater than that of other vector mosquitoes such as *Cx. quinquefasciatus* and *An. gambiae*, (e.g., Chen et al., 2015; Olson and Bonizzoni, 2017; Palatini et al., 2017). These findings have led to some speculation that the mechanism for vpiRNA biogenesis during arbovirus infection involves mosquito genome-integrated viral sequences as templates. However, there are at least two arguments against inherited EVEs serving as templates for vpiRNAs during acute infection: 1) integration of arboviral viral-derived cDNA (vDNA) into the chromosome to form a heritable EVE would require arboviral infection of the mosquito germline (ovarian) tissue, and this is infrequently observed for any except bunyaviruses (Watts et al., 1973; Lumley et al., 2017) and less frequently flaviviruses (Aitken et al., 1979; Sánchez-Vargas et al., 2018); and 2) the vast majority of characterized EVE sequences in *Aedes* spp. mosquitoes are homologous to the genomes of ISVs rather than to circulating arboviruses and thus do not appear to have been recently integrated. An interesting exception is the integration of large segments of DENV vDNA found in Foshan strain *Ae. albopictus* populations from southeast China (Chen et al., 2015). Additionally, since the length(s) over which piRNAs and their targets must have complementarity for effective cleavage has not been determined, imperfect matches between EVE-derived piRNAs that are related to arboviruses and viral genome RNAs might be tolerated.

Nevertheless, short DNA sequence fragments homologous to the genome of an infecting arbovirus have been detected in total DNA extracted from *Aedes* spp. and *Culex* spp. mosquitoes or cell cultures infected by alphaviruses, flaviviruses, and bunyaviruses (Goic et al., 2016; Nag et al., 2016; Nag and Kramer, 2017; Rückert et al., 2019) (see **Tables 1** and **2**). The reduction in synthesis of these “vDNAs” by treatment with reverse transcriptase inhibitors (Goic et al., 2016) suggests that their mechanism of biosynthesis is through recombination of an infecting viral RNA genome with a retrotransposon transcript during its reverse transcription in the cytoplasm and incorporation of the vDNA into a hybrid extrachromosomal element (Goic et al., 2016). It has not been determined whether the vDNAs detected in infected cells remain extrachromosomal or if any have been integrated into the mosquito genomes, or whether they serve as templates for biogenesis of vpiRNA precursors. That is, there has been no direct demonstration of the entire pathway from synthesis of vDNA to its integration into the mosquito genome to its use as a template for transcription of precursor vpiRNA.

### Which Members of the Expanded PIWI Family Genes of *Aedes* spp. Mosquitoes Have Been Implicated in Production of vpiRNAs? How?

Studies to determine which PIWI family protein(s) is/are involved in vpiRNA biogenesis have largely employed transient knockdown of expression of *ago3* or *piwi1-7* mRNA by transfection of mosquito cell cultures with dsRNA homologous to the mRNA under study. Schnettler et al. (2013) showed that knockdown of Piwi4 expression in Aag2 cells resulted in enhanced replication of SFV (*Alphavirus*), while knockdown of other Piwi protein expression affected virus replication only slightly; however, expression of SFV-specific vpiRNAs in the same cells was altered very little by knockdown of Piwi proteins. In contrast, Miesen et al. (2015) found that knockdown of either Ago3 or Piwi5 greatly reduced expression of SINV (*Alphavirus*)-specific vpiRNAs in Aag2 cells and Piwi4 or Piwi 6 knockdown had a much lesser effect. Positive (genome)-sense vpiRNAs were bound by Ago3, and Piwi5-bound antisense vpiRNAs in the cytoplasm (Miesen et al., 2015). Varjak et al. (2017b), in an examination of SFV-infected Aag2 cells, confirmed the reduction of alphavirus-derived vpiRNA levels after Ago3 and Piwi5 knockdown. In DENV (*Flavivirus*)-infected Aag2 cells, Ago3 and Piwi5 were also found to be essential for vpiRNA generation, with Piwi6 involved to a lesser extent; however, DENV vpiRNAs were exclusively positive (genome) sense (Miesen et al., 2016a). Varjak et al. (2017b), in a further exploration of the role of Piwi4, showed that it associates with Ago3, Piwi5, and Piwi6, as well as with Ago2 and Dcr2, which are part of the exo-siRNA pathway, in SFV-infected Aag2 cells and that Piwi4, probably in complex with one of the above proteins, also associated with virus-derived small interfering RNAs (vsiRNAs).

Other *Ae. aegypti* cell culture proteins, such as the Tudor protein Veneno, also have been implicated in the non-canonical expression of vpiRNAs during infection by an alphavirus (Joosten et al., 2018).

Thus, in *Ae. aegypti* Aag2 cells infected with both alphaviruses and a flavivirus, reduction in the expression levels of Ago3 and Piwi5 has been shown to reduce the production of vpiRNAs. The roles of other members of the expanded *Ae. aegypti* PIWI family proteins, particularly Piwi4, in the production of vpiRNAs and involvement in piRNA-mediated antiviral defense are complex and might vary with the arbovirus–mosquito combination. Studies to date have been carried out only in mosquito cell cultures, and whether or not transient reduction of expression of any one of *Ae*. *aegypti* PIWI family proteins in adult mosquitoes affects alphavirus or flavivirus replication remains an open question, as does a possible link between the piRNA and exo-siRNA pathways.

## Points to Ponder in Attempting to Deduce the Role of vpiRNA Production in the Vector Mosquito Antiviral Immune Response in the Mosquito–Arbovirus Arms Race

Use of mosquito cell cultures has allowed molecular and genetic characterizations that would be more difficult in mosquitoes, but use of cultured cells derived from embryonic or larval stages of development, with unknown tissues of origin, as surrogates for the variety of tissues found in adult female mosquitoes has limitations.

The notion that the vpiRNA pathway might be a part of antiviral defense resulted from several observations.

Although it had been amply demonstrated that the exo-siRNA pathway is the major vector mosquito antiviral response, the observation that excessive levels of vpiRNAs were produced after infection of exo-siRNA-defective cultured cells led to our original speculation that production of vpiRNAs in mosquito cells in the absence of vsiRNAs might serve a compensatory antiviral role. The three exo-siRNA-incompetent *Aedes* spp. cell culture lines identified to date all appear to produce vpiRNAs in excess after both arbovirus and ISV infections. Interestingly, these cell lines have never been observed to be naturally persistently infected with ISVs.

Observations that vpiRNAs arise during many acute arbovirus infections in exo-siRNA-competent *Aedes* spp. mosquitoes and cultured cells, coupled with knowledge that the piRNA pathway serves as a defense system against transposon activity in *Drosophila*, added to speculation that the expanded number of PIWI family genes in mosquitoes might code for functions in addition to genome defense from transposons. Finding of EVEs in piRNA clusters that could serve as progenitors of vpiRNAs helped build a hypothetical pathway to the antiviral role of the mosquito piRNA pathway.

The piRNA pathway in vector mosquitoes is not well understood, and although research on the functions of the mosquito piRNA pathway has been active recently, knowledge is not yet sufficient to completely answer several of the questions posed in this review.

Observations and evidence to suggest that the vector mosquito piRNA pathway is not an obligate or essential antiviral defense response to acute arbovirus infections also have been put forward.

The demonstration that some natural arbovirus–vector combinations (largely those involving *Culex* spp. vectors) do not result in the production of vpiRNAs suggests that the piRNA pathway is not an essential component of anti-arboviral defense in all vector mosquitoes. Further, it is clear that the vpiRNA response of the other major genus of arbovirus vectors, the *Aedes* spp. mosquitoes, varies in magnitude and pattern of vpiRNAs produced depending on which of the most prevalent arbovirus families, e.g., flaviviruses, alphaviruses, and bunyaviruses, has infected the mosquito. In other words, “one size fits all” does not apply in describing the vector mosquito vpiRNA response to arbovirus and ISV infections.

In addition to findings that *Culex* spp. vectors are less likely than *Aedes* spp. vectors to produce vpiRNAs, several other interesting comparisons between the two genera can be made.

A far lower proportion of *Culex* spp. vector genomes is occupied by repeat elements, including EVEs, than *Aedes* spp. (Chen et al., 2015). Many arboviruses transmitted by *Culex* spp., particularly encephalitic alphaviruses and flaviviruses, are not vertically transmitted and are more likely to cause cytopathology in tissues of their natural vector (Weaver et al., 1992; Girard et al., 2005). The question arises whether or not comparative cytopathology of arboviruses in their natural *Culex* spp. vectors (e.g., encephalitic alphaviruses and flaviviruses) is related to vpiRNA production and role. The failure to find EVEs related to potentially cytopathogenic viruses in the genomes of vector mosquitoes and the lack of ability of these viruses to undergo vertical transmission in their vectors could all be factors in whether vpiRNAs are generated and their roles in the RNA-based immunity of natural vector mosquitoes for these viruses.

However, *Culex* spp. vector mosquitoes and cultured *Cx. tarsalis* CT cells have been shown to harbor persistent ISV infections, which are vertically transmitted in the case of mosquitoes. Cultured *Culex* spp. cells have also been shown to generate vpiRNAs in response to persistent infection with certain ISVs. The consistent finding of ISV vpiRNAs during persistent infection of both *Culex* and *Aedes* spp. mosquitoes suggests that control of persistent virus infections is a major function of the expanded PIWI family genes in vector mosquitoes.

To date, it has been difficult to determine a consistent pattern for the mosquito PIWI family proteins involved in the biogenesis of vpiRNAs during either arbovirus or ISV infections, even in a single type of cultured cells, such as *Ae. aegypti* Aag2 cells. Differences in magnitude of the piRNA response, possession by vpiRNAs of the ping-pong amplification signature, as well as whether or not vpiRNAs are produced at all suggest there are large differences between virus families, even in the same mosquito vector. This observation suggests different mechanisms of vpiRNA biogenesis for different virus families, perhaps due to differences in viral genome organizations, patterns of replication, and abundance of templates or triggers for the response. Finally, deducing the template for transcription of vpiRNA precursors is an intriguing exercise. Although it is clear that some piRNAs are derived from non-retroviral RNA virus sequences integrated into genomes, which occur naturally and abundantly in *Ae. aegypti* and *Ae. albopictus*, and that these mosquitoes are capable of generating vDNA from genomes of certain arboviruses during acute infection, no direct path between newly generated vDNA, EVEs, and vpiRNAs has yet been demonstrated.

Hypothetically, many of the remaining questions regarding the biogenesis and role of virus genome-derived PIWI-associated RNAs (vpiRNAs) in mosquito antiviral defense could be answered in a study in which vector mosquitoes from both *Aedes* and *Culex* genera would be freshly captured from the wild and colonized in the laboratory. They should be characterized with regard to natural ISV infections and preexisting EVEs in their genomes. Then, each would be orally infected with newly isolated, low-passage representatives of alpha-, flavi-, and bunyavirus families (preferably more than one representative from each family that is naturally transmitted by the vector genus infected). Over the time course of the infection, mosquitoes would be monitored for the production of sRNAs, vDNAs, and virus replication and transmission. Infected mosquitoes would be allowed to reproduce and their progeny monitored for vertical transmission of the acutely infecting arbovirus and development of new EVEs. It may be necessary to continue the study through several mosquito generations to observe development of EVEs that generate vpiRNAs related to the originally infecting virus. Appropriate controls would of course be required, and mosquitoes could be genetically manipulated to eliminate expression of each PIWI family protein to hopefully tease out their roles in vpiRNA biogenesis.

Perhaps such patient, long-term, multigenerational studies with adult female mosquito vector–arbovirus combinations with consideration of the virus tissue tropisms and ability to undergo vertical transmission will be the key to better understanding of the role of the piRNA pathway in vector mosquito antiviral immunity.

## Author Contributions

The author confirms being the sole contributor of this work and approved it for publication.

## Conflict of Interest

The author declares that the research was conducted in the absence of any commercial or financial relationships that could be construed as a potential conflict of interest.

The handling editor declared a past co-authorship with the author.
